# Production of polyhydroxybutyrate in oil palm (*Elaeis guineensis* Jacq.) mediated by microprojectile bombardment of PHB biosynthesis genes into embryogenic calli

**DOI:** 10.3389/fpls.2015.00598

**Published:** 2015-08-11

**Authors:** Ghulam Kadir Ahmad Parveez, Bohari Bahariah, Nor Hanin Ayub, Mat Yunus Abdul Masani, Omar Abdul Rasid, Ahmad Hashim Tarmizi, Zamzuri Ishak

**Affiliations:** Advanced Biotechnology and Breeding Centre, Malaysian Palm Oil BoardKajang, Malaysia

**Keywords:** oil palm, monocot, transgenic, biolistics, biodegradable plastics

## Abstract

Biodegradable plastics, mainly polyhydroxybutyrate (PHB), which are traditionally produced by bacterial cells, have been produced in the cells of more than 15 plant species. Since the production of biodegradable plastics and the synthesis of oil in plants share the same substrate, acetyl-coenzyme A (acetyl-CoA), producing PHB in oil bearing crops, such as oil palm, will be advantageous. In this study, three bacterial genes, *bktB*, *phaB*, and *phaC*, which are required for the synthesis of PHB and selectable marker gene, *bar*, for herbicide Basta resistant, were transformed into embryogenic calli. A number of transformed embryogenic lines resistant to herbicide Basta were obtained and were later regenerated to produce few hundred plantlets. Molecular analyses, including polymerase chain reaction (PCR), Southern blot, and real-time PCR have demonstrated stable integration and expression of the transgenes in the oil palm genome. HPLC and Nile blue A staining analyses confirmed the synthesis of PHB in some of the plantlets.

## Introduction

Oil palm is a major economic crop for Malaysia. Area planted with oil palm has increased from 55,000 hectares in 1960 to 5.39 million hectares by end of 2014 (Anon, 2015). However, lately the area cultivated with oil palm has been almost stagnant due to unavailability of arable lands as well as Malaysia’s desire to keep its forest and maintain its reputation as one of the 12 mega biodiversity countries. Since 2007, palm oil has become the largest source of edible oil in the world. World palm oil is mainly produced from plantations in Malaysia and Indonesia. In order to maintain its premier position and to remain competitive, threats such as shortage of labor and arable land and fluctuation in commodity price need to be overcome by increasing yield per unit area as well as producing novel high value products using approaches such as genetic engineering (Parveez et al., 2015). From previous experience, it was estimated that 4–5 years are required to produce transgenic oil palm plantlets from tissue culture explants (Parveez et al., 2000).

The world’s first transgenic plant, tobacco (Fraley et al., 1983) was produced more than 30 years ago. Since then, the number of transgenic plant species developed has been increasing. Recent report by the International Service for Acquisition of Agri-biotech Applications (ISAAA) shows that the areas commercially planted with transgenic plants worldwide have been increasing annually, from 1.7 million hectares in 1996 to 181.5 million hectares in 2014 (James, 2014). The acreage reported is contributed by 28 countries and involves mainly soybean, maize, and cotton. Nevertheless, other crops such as papaya, squash, canola, sugar beet, sweet pepper, and alfalfa are also actively being planted and contribute to the statistics.

Genetic modification, is a method for the production of environmentally adaptive crops, higher value metabolites and economical important traits such as oils and industrial feedstock. Biodegradable plastics, or bioplastics, are one such product which has great potential and high demand. One example of biodegradable plastics is polyhydroxybutyrate (PHB), which is also the most common polyhydroxyalkanoate or PHA. PHB is normally produced by bacteria as a storage material under restricted growth conditions (Senior and Dawes, 1973). Even though PHA was discovered ∼90 years ago, only in the recent decades it has been recognized for its thermoplastic and elastomeric properties (Poirier, 2002). Due to its inherent characteristics, bioplastics can be completely degraded, under optimal conditions, to CO_2_ and H_2_O (Lőssl et al., 2003). It is a useful polymer which could be exploited to produce a wide range of environmentally friendly industrial polymers. PHB is a stiff and relatively brittle polymer in nature (Holmes, 1988) and it has been reported to have chemical and physical properties similar to polypropylene (Steinbüchel, 1991).

In depth understanding of the PHB synthesis process, has led to commercial production of PHB. Bacterial fermentation was initially used in 1980s to commercially produce PHA from *Ralstonia eutropha.* However, the commercialization was on limited scale due to high cost of production, especially the need to supply costly substrates (Anderson and Dawes, 1990). It was recently reported that PHA produced by a US based company was marketed at USD 4.96–6.06/kg as compared to propylene which has comparable properties but non-biodegradable and selling at USD 1.65/kg (Agnew and Pfleger, 2013). The high cost of production of PHA has forced scientists to explore alternative approaches to produce it at a lower price. Poirier (2002) has proposed plants as potential system as plants are capable of producing millions of tons of oils and starch at a lower cost of between USD 0.25 and 1.0/kg.

**FIGURE 1:**
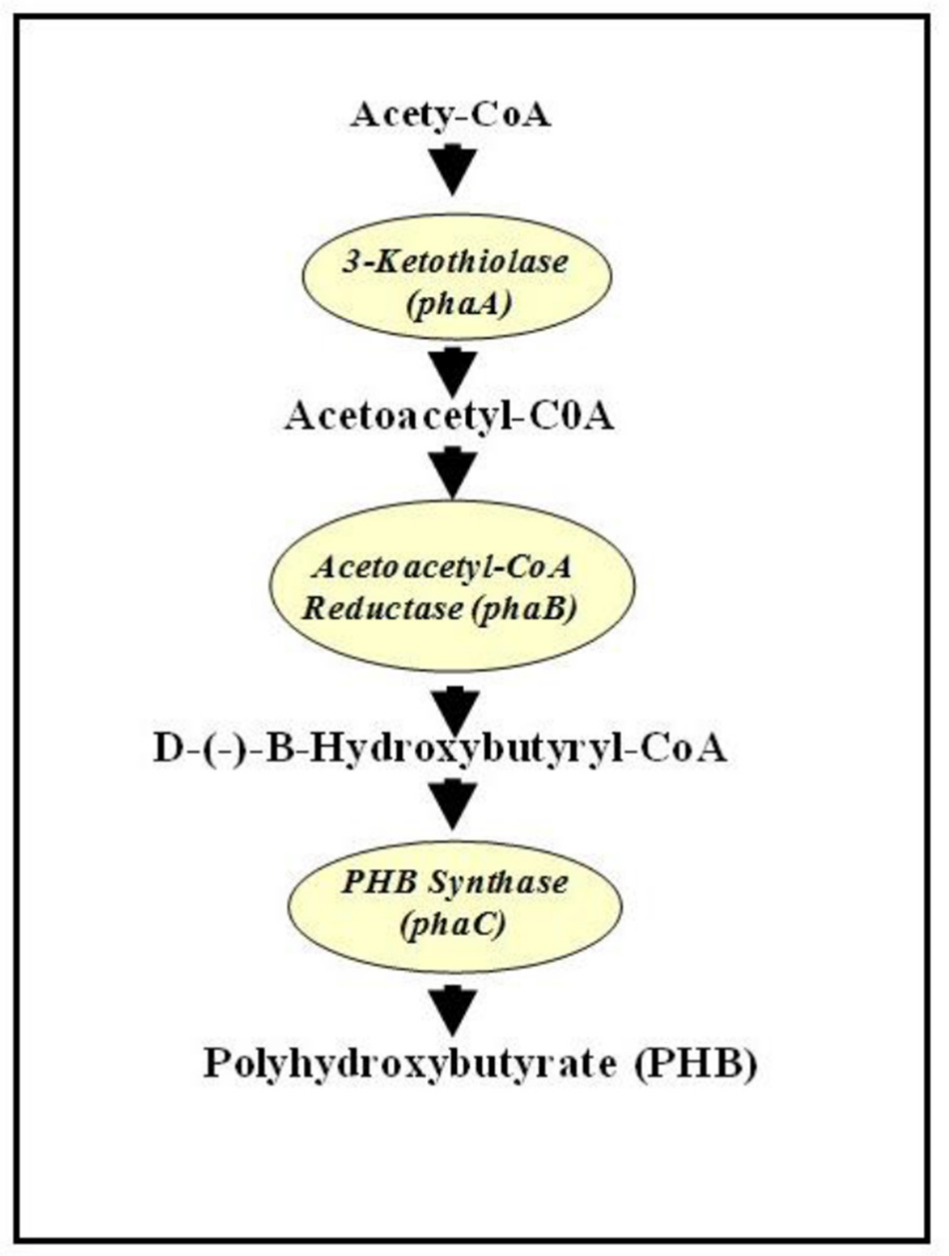
**Biosynthesis pathway of polyhydroxybutyrate (PHB) from acetyl-CoA.** Adapted from Poirier (2002).

It was shown that in bacterial system, PHB is synthesized from acetyl-CoA following three enzymatic reactions. The first enzyme, 3-ketothiolase (phaA or bktB), catalyzes the reversible condensation of two acetyl-CoA moieties to form acetoacetyl-CoA. Acetoacetyl-CoA reductase (phaB) subsequently reduces the acetoacetyl-CoA to D-(-)-3-hydroxybutyryl-CoA, which is then polymerized by PHB synthase (phaC) to produce PHB (Anderson and Dawes, 1990) (**Figure 1**). In plant system acetyl-CoA are found in the following organelles: cytosol, plastid, mitochondria, and peroxisome. Therefore, Poirier (2002) has postulated that theoretically, PHB could also be synthesized in any of those sub-cellular compartments in plants. First demonstration of PHB production in plant was in *Arabidopsis* cytoplasm with a maximum of 0.1% dry weight (dwt) of PHB (Poirier et al., 1992). Judging from low level of PHB synthesis in cytoplasm, plastid was later targeted for accumulating PHB as it is the organelle for fatty acid synthesis and has the highest flux of acetyl-CoA, also the substrate of PHB synthesis (Nawrath et al., 1994a). When all the PHB genes (on individual plasmids and involving crosses) were targeted into the plastid of *Arabidopsis*, up to 14% dwt of PHB was reported (Nawrath et al., 1994b). When all the PHB genes were fused onto a single plasmid, up to ∼40% dwt PHB was produced, the downside being that the plants were extremely stunted and chlorotic (Bohmert et al., 2000). Later, when the PHB genes were induced by a chemical, methoxyfenozide, up to 14.3% dwt PHB was produced without any deleterious effects on the plants (Kourtz et al., 2007). Targeting PHB into plastid of poplar tree and induced by ecdysone resulted in around 3.69% dwt PHB. However, it was observed that plants with 1% dwt PHB and higher showed negative growth characteristics (Dalton et al., 2011). In rapeseed around 3–7% dwt of PHB was obtained when PHB genes were targeted to the plastid (Houmiel et al., 1999). In potato, when the *phaA* gene was induced chemically, PHB was synthesized in leaves at a rate of up to 0.009% dwt (Bohmert et al., 2002). Recently, up to 13.7% dwt of PHB was synthesized in T4 seeds of camelina when the PHB genes were targeted to the plastid and driven by seed-specific promoter (Malik et al., 2015).

In sugarcane, when the PHB genes were targeted into the cytosol, mitochondria or plastid, synthesize was observed only in plastid at up to 1.88% dwt, with no agronomic penalty (Petrasovits et al., 2007; Purnell et al., 2007). Testing different promoters resulted in up to 4.8% dwt PHB being produced in sugarcane but with reduced biomass and slight chlorosis (Petrasovits et al., 2012).

Besides cytosol and plastid, PHB was also reported to be synthesized in other organelles such as peroxisomes in Black Mexican Sweet maize [2% fresh weight (FW)], leucoplasts of sugar beet hairy root (5.5% dwt); stem of flax (0.005% FW) and in seed coat/vacuole of soybean (0.36% dwt; Hahn et al., 1999; Menzel et al., 2003; Wrobel-Kwiatkowska et al., 2007; Schnell et al., 2012). It was also reported that when the PHB genes were transformed using plastid transformation method into tobacco, up to 18.8% dwt of PHB was reported (Bohmert-Tatarev et al., 2011). However, even though for the plants which were fertile and produced viable seed, delayed flowering was observed.

As oil palm is an oil bearing crop, acetyl-CoA pool is expected to be high and potential target for synthesizing PHB. In this paper, we describe the transformation of oil palm embryogenic calli with PHB genes driven by maize ubiquitin promoters and targeting it to plastid. The three genes used for synthesizing PHB were from the bacteria *Alcaligenes eutrophus* H16 (Peoples and Sinskey, 1989a,b,c). The research was carried out at the laboratory scale and regenerated transgenic oil palms are planted in a fully contained biosafety greenhouse for evaluation. This is the first report demonstrating the successful synthesis of PHB in transgenic oil palm plantlets. Even though there were earlier reports on the attempt to produce transgenic oil palm carrying PHB genes (Parveez et al., 2008; Ismail et al., 2010), however, none of them provide any evidence that PHB was synthesized in the transgenic plantlets. At the same time efforts to produce newer transformation vectors with combination of various constitutive promoters as well as specifically targeting the PHB genes into oil palm leaves has also been reported (Yunus et al., 2008; Masani et al., 2009).

## Materials and Methods

### Oil Palm Embryogenic Calli Transformation

One micron sized gold microcarriers were precipitated with pME22 plasmid (Willis et al., 2008) according to the manufacturer’s instructions (Bio-Rad Laboratories for Biolistics PDS/He 1000). Bombardment was carried out as described by Parveez et al. (1997, 1998). After bombardment, the embryogenic calli were maintained on solid MS macro and micronutrients supplemented with 2.2 mg/l 2,4-D and 30 g/l sucrose (pH 5.7) and cultured at 28°C, in the dark, and subcultured every 30 days onto fresh medium.

### Regeneration of Transformed Embryogenic Callus

Transformed embryogenic calli were selected from non-transformed cells by exposing them to a selection medium containing 50 mg/l Basta (Parveez et al., 1996, 2007) for 3 weeks post-bombardment. The transformed embryogenic calli were subcultured onto fresh medium containing the same concentration of selection agent at monthly intervals. The selected cultures were later regenerated into polyembryogenic cultures, small plantlets, and finally producing roots on various media following the procedure described by Parveez et al. (2000). The fully regenerated and hardened plantlets were transferred into polybags and grown in a biosafety screenhouse.

### Plant Total DNA Isolation

Large scale plant total DNA extraction was carried out according to the modified CTAB method (Doyle and Doyle, 1987). Ten grams of leaves were ground in liquid nitrogen and later added with 10 ml CTAB extraction buffer (100 mM Tris-HCl; pH 8.0; 20 mM EDTA; 1.4 M NaCl; 2% CTAB; 1% PVP; and 0.2% 2-mercaptoethanol) and incubated for 1 h at 65°C. Ten-milliliters of chloroform: isoamyl alcohol (24:1) were added after cooling for 20 min at room temperature, and centrifuged at 4°C, 20,000 *g* for 5 min. Aqueous extract was cleaned twice with 5 ml of chloroform: isoamyl alcohol (24:1) prior to DNA precipitation by adding 6 ml of chilled isopropanol. The extract was centrifuged at 20,000 *g* for 5 min after precipitation for 20 min at room temperature. Pellet was suspended (76% ethanol, 1 mM ammonium acetate), incubated for 20 min at room temperature and centrifuged at 20,000 *g* for 5 min. Final pellet was dissolved in 500 μl TE Buffer.

### Polymerase Chain Reaction (PCR)

Amplification of *bar*, *bktB*, *phaB*, and *phaC* gene was carried out using standard or touch-down PCR protocols (Sambrook et al., 1989). Fifty-nanograms of oil palm DNA and 1 ng of plasmid DNA were used in the PCR reactions. The following amplification condition was used in the standard procedure: 30 cycles at 92°C (50 s), 60°C (50 s), and 72°C (60 s). For the *bar* gene specifically, the touchdown procedure, 10 cycles 92°C (45 s), 70°C (45 s; -0.5 C per cycle), 72 C (60 s), and 20 cycles 92 C (45 s), 65 C (45 s), and 72 C (60 s) was used. Amplified PCR fragments were resolved by electrophoresis on 1.4% agarose gels in 0.5X TBE (45 mM Tris-Borate; 1 mM EDTA, pH 8.0) buffer.

### Southern Blot Hybridization

Twenty-micrograms of undigested and overnight *Eco*RI digested transformed and untransformed oil palm DNA were separated on 1.0% agarose gels and later transferred onto nylon membranes (Hybond-N, Amersham) using a vacuum pump at 55 mbar pressure. *Bar* gene fragment (361 bp) was labeled with DIG (Roche Molecular Biochemicals) according to the manufacturer’s instructions. Labeled probes were then hybridized to the membrane at 65°C. The membranes were washed twice with 2X washing solution (5 min each) and twice with 0.5X washing solution (15 min each at 65°C). The membranes were later blocked with a blocking reagent and incubated with anti-DIG to bind the antibody conjugates to the labeled DNA. The bound antibody was detected by using a chemiluminescent assay (CSPD). The membranes were exposed to film at room temperature for 1–2 h.

### Total RNA Extraction

A method modified from Zeng and Yang (2002) was used to isolate RNA from transgenic samples. Ten grams of frozen leaf tissues were ground into powder in a mortar in the presence of liquid nitrogen and later transferred to a 30 mL extraction buffer [0.05 M Tris-HCl (pH 8.5), 0.15 M LiCl, 5 mM EDTA, 5% SDS, 0.1 M aurin tricarboxylic acid, 0.4% β-mercaptoethanol] in 50 mL centrifuge tube (SS34). Fifteen-milliliters each of phenol (pH 8.0) and chloroform were added to the homogenate and the phases were separated by centrifugation (20,000 *g*, 25°C, and 30 min). The aqueous layer was removed to new centrifuge tubes and re-extracted with addition of 15 mL each of phenol (pH 8.0) and chloroform. The aqueous layer was later added with equal volume of chloroform:isoamylalcohol (24:1), vortexed and centrifuged at 20,000 *g* for 30 min at 25°C. Eight molar LiCl was added to the aqueous layer to make a final concentration of 2 M. The mixture was mixed by inversion and incubated overnight at 4°C to precipitate the RNA. The RNA was pelleted by centrifugation at 20,000 *g* (4°C) for 30 min and resuspended in 1.5 ml of 2 M LiCl. After another round of centrifugation at 20,000 *g* for 30 min, the pellet was dissolved in 5 ml of RNase-free water. Eight molar of LiCl was again added to the mixture to a final concentration of 2 M, mixed and stored at 4°C overnight to precipitate the RNA. The RNA was pelleted by centrifugation at 20,000 *g* (4°C) for 30 min, rinsed with 4 ml of 2 M LiCl, and resuspend in 1 ml RNase-free water prior to centrifugation at 12,000 *g* for 5 min to pellet insoluble materials. The supernatant was transferred to a new SS34 tubes and 1/19 volumes of 3 M sodium acetate (pH 5.2) and 2.5 volumes of absolute ethanol were added to the mixture. The mixture was mixed and stored at -80°C for at least 2 h to precipitate the RNA. The RNA was pelleted by centrifugation at 20,000 *g* (4°C) for 10 min. The supernatant was discarded and the pellet was rinsed two times in 1 ml 70% cold ethanol and dried under vacuum. The RNA was dissolved in RNase-free water and stored at -80°C until required.

### Real Time PCR

Total RNA clean-up was carried out using the Qiagen RNase-free DNase kit according to the manufacturer’s protocol to remove DNA and other impurities. Concentration and purity of the RNA were determined using the NanoDrop ND-1000 Spectrophotometer. Three-hundred nanogram per microliters of cleaned total RNA was subjected to integrity analysis using the Agilent 2100 Bioanalyzer (RNA 6000 Nano Assay Kit). Intact RNA was converted to cDNA by using the High Capacity cDNA Archive Kit (Applied Biosystems). Real-time PCR was carried out with 10 μl 2x TaqMan Universal PCR Master Mix, 1 μl 20x Assay Mix (containing specific primers and probe) and 9 μl cDNA (diluted in RNase-free water). Gene fragment was used as a control of the specificity of primers and probes used in the amplification. PCR cycling parameters were 50°C for 2 min, 95°C for 10 min, and 40 cycles of 95°C for 15 s and 60°C for 1 min. Real-time detection of fluorescence was performed on the ABI PRISM 7000 Sequence Detection System (Applied Biosystems, USA). To determine the relative expression level of the transgenic lines, the average Cq value of the transgene was normalized to the average *C*q value of *GAPDH* (endogenous control) and then compared to the calibrator. For this experiment, untransformed oil palm was used as the calibrator. Relative quantification (RQ) of the gene expression was calculated using the RQ Study Application in the 7000 System SDS Software version 1.2.3 (Applied Biosystems, USA) which was based on the comparative ddC_T_ method (Livak and Schmittgen, 2001).

The efficiency of the TaqMan reaction was determined by the method described by Toplak et al. (2004). A five serial 10-fold dilutions of a positive control template was carried out and the Cq values was plotted as a function of log_10_ concentration of template. The slope of the resulting line is a function of the PCR efficiency. The PCR efficiency was calculated by putting the slope (*S*) value into the following equation: PCR efficiency (%) = {[10(1/-*S*)]-1}× 100. cDNAs generated from the *R. eutropha* were used to generate standard curve for all the PHB genes while cDNAs generated from untransformed oil palm were used to generate the standard curve for the *GAPDH* gene.

### High Performance Liquid Chromatography Analysis

Detection of PHB in the transformed oil palm was carried out using HPLC. The presence of the PHB in the samples was measured using an acidic methanolysis and hydrolysis method according to Karr et al. (1983). Two gram leaf samples were dried in an 80°C oven. The dried samples were ground to powder in liquid nitrogen. The powdered samples were transferred into glass tubes and 1 ml concentrated H_2_SO_4_ was added prior to incubation at 90°C for 30 min. This acid-treatment step depolymerized the PHB by elimination of water to yield crotonic acid (trans 2-butenoic acid). After incubation, the reaction mixture was cooled on ice. This was followed by addition of 4 ml 0.014 N H_2_SO_4_. After thorough mixing, the sample was filtered into a new glass tube through a LC 13 PVDF 0.2 μm membrane. Then, 15 μl of the filtered sample were transferred into appropriate vials containing 135 μl 0.014 N H_2_SO_4_ for HPLC analysis. Detection of crotonic acid was performed at 210 nm.

### Nile Blue A Staining Method

Nile blue A staining was carried out using a protocol by Ostle and Holt (1982) with some modifications. A 1% (v/v) aqueous solution of Nile blue A (Sigma) was prepared by heating at 50°C to dissolve the stain, and then filtered before use. Leaf samples were cut into small square sections and were stained with 500 μl Nile blue A solution at 55°C for 10 min on slide. The slides were washed with sterile water and then with 8% (v/v) aqueous acetic acid. The slides were washed again with sterile water, blotted dry with 3 MM whatman paper, and then covered with a glass cover slip. The stained samples were viewed under an excitation wavelength of 460 nm by using Leica stereomicroscope (Model MZ12.5). A fluorescence Plus filter module (Leica) was used to reduce autofluorescence from chlorophyll.

## Results and Discussions

### Regeneration of Transgenic Oil Palm Plantlets

**FIGURE 2:**
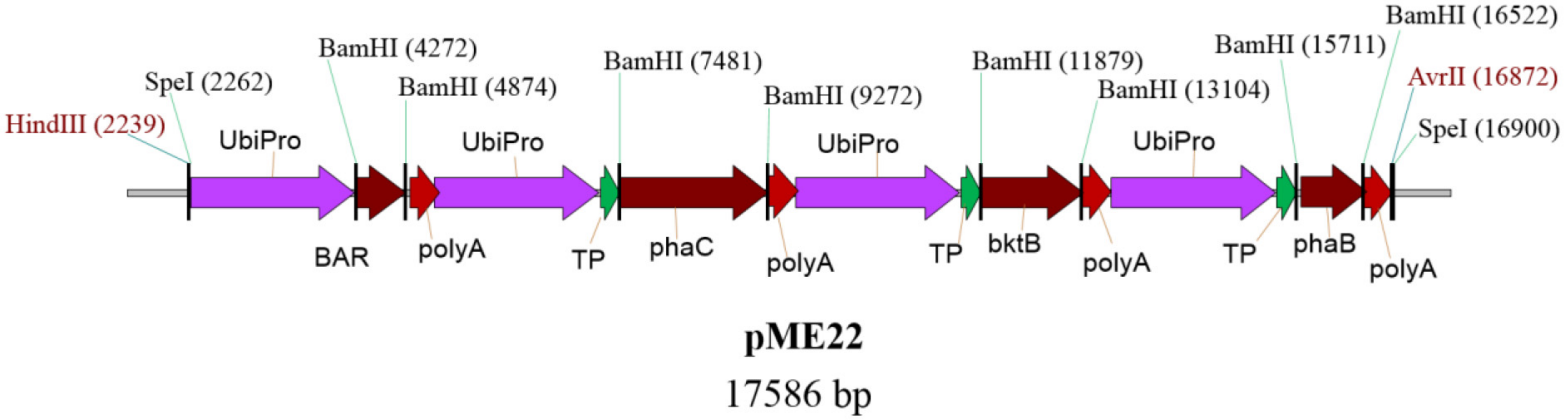
**Schematic diagram of plasmid pME22 carries bar, *phaC*, *bktB*, and *phaB* genes driven by maize ubiquitin promoter.** All the *phaC, bktB*, and *phaB* genes were fused at the 5′ end with a transit peptide of small subunit of rubisco from pea. The full size of the pME22 plasmid is 17,586 bp. UbiPro, maize ubiquitin promoter; BAR, gene for phosphinothricin acetyltransferase; poly A, 7S 3’ beta conglycinin transcriptional termination sequence; TP, transit peptide of small subunit of rubisco; bktB, β-ketothiolase; phaB, acetoacetyl-CoA reductase; phaC, PHA synthase.

Oil palm embryogenic calli were transformed with the plasmid pME22, carrying *bar*, *bktB, phaB*, and *phaC* genes using microprojectile bombardment based on optimized parameters (Parveez et al., 1997, 1998). A schematic representative of plasmid pME22 is given in **Figure 2**. Embryogenic calli bombarded without DNA was used as a negative control. All the three genes for synthesizing PHB were individually inserted with a transit peptide of the small subunit of rubisco from pea (Nawrath et al., 1994b). Maize ubiquitin promoter and its intron (Christiensen et al., 1992) was used to drive all the PHB genes and selectable marker gene (*bar*) as it has been proven to be the best promoter for expressing transgenes in oil palm (Chowdhury et al., 1997). All the four genes used in this study were fused together into one vector sequence because it was shown in *Arabidopsis* that it could result in higher accumulation of PHB (Valentin et al., 1999; Bohmert et al., 2000; Mitsky et al., 2000). This could also help ensure that all the genes are closely integrated into the genome and allow for higher gene expression and reduce gene silencing (Mitsky et al., 2000).

Oil palm embryogenic calli were initially cultured on a medium without selection agent for ∼3 weeks. This was followed by a step-by-step (two stages) selection approach with the hope of producing a higher number of transformants and also overcoming the regeneration difficulties. The transformed embryogenic calli were subcultured onto fresh medium containing selection agent, once a month. Initial selection was carried out by exposing the bombarded embryogenic calli to half strength of selection agent (25 mg/l). The selected transgenic embryogenic calli were later subcultured onto fresh medium containing full strength of the selection agent (50 mg/l). It was observed that upon transfer to fresh medium containing Basta, untransformed embryogenic calli began to die and allowing only resistant embryogenic calli to proliferate selectively. Generally, Basta resistant embryogenic callus colonies started emerging after 6–8 months on selection medium.

**FIGURE 3:**
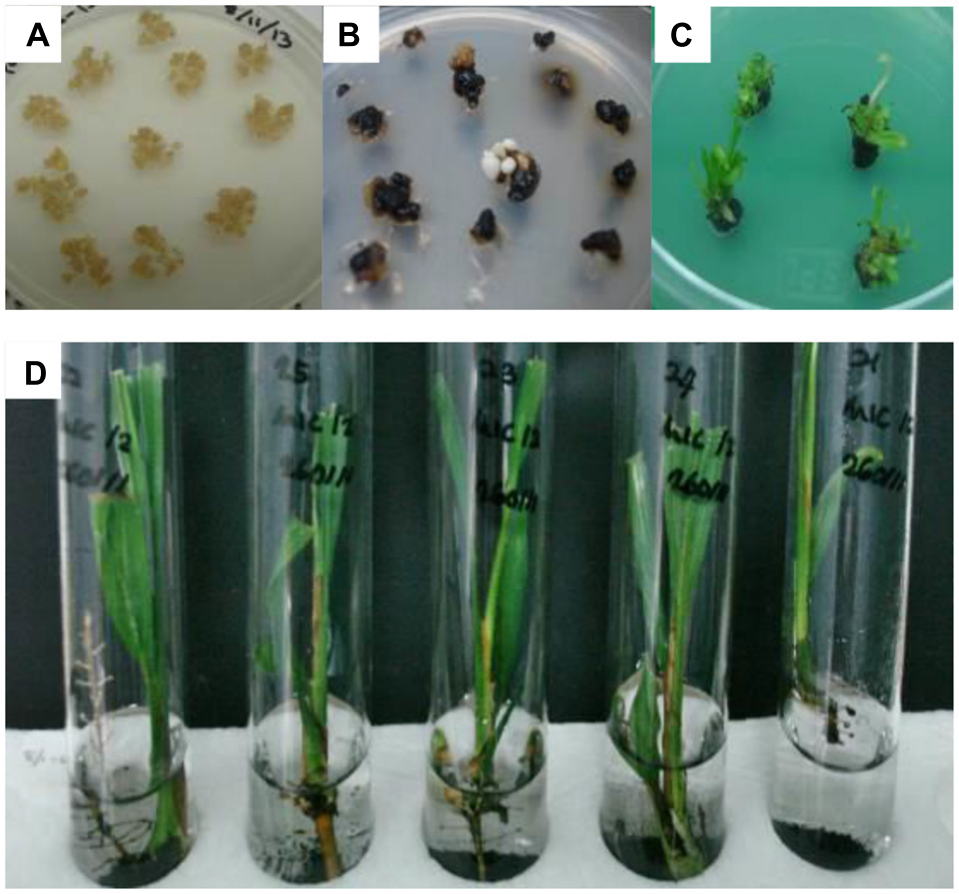
**Regeneration process of oil palm plantlets. (A)** Embryogenic calli before bombardment; **(B)** Transformant calli surviving on selection media; **(C)** Shoot development on selection media; and **(D)** Transformed plantlets with roots.

The freshly emerged Basta resistant embryogenic calli were proliferated on the same medium containing selection agent until the size of colony became bigger and turned into embryoids. The transgenic embryoids began to regenerate on the selection medium where the whitish embryoids became greenish (polyembryogenic) after 3–5 months of culture on polyembryogenic inducing medium. After 2–3 months, some of these polyembryogenic cultures started to produce shoots. Once these shoots were big enough, they were individually isolated from the polyembryoids cultures and transferred onto conical flasks or test tubes containing shoot inducing medium for shoot elongation. After ∼2–3 months the elongated shoots were transferred into test tubes containing root inducing liquid medium for further development and root initiation. After ∼2 months in liquid root inducing medium, individual plantlets, with good rooting system were obtained. The plantlets were transferred onto soil in small polybags and grown in a biosafety screenhouse (**Figure 3**). These plantlets were fertilized and maintained according to standard nursery practices for oil palm. All plantlets showed normal phenotype and growth characteristics.

The initial culturing of bombarded tissues on embryogenic calli medium in the absence of selection agent for 3–4 weeks is to allow transformed cells to divide several times prior to selection. The cell division process will result in a critical mass of transformed cells which is important for the survival of cells under selection pressure (Ozias-Akins et al., 1993). Selection using a lower concentration of Basta is preferable as there is some evidences in other systems suggesting that regeneration capacity of stably transformed embryogenic calli increases under these conditions. It was reported earlier that selection of transformed rice at a lower concentration of hygromycin (50 mg/l as compared to 100 mg/l) resulted in a higher number of transformed calli and transgenic plants (Christou and Ford, 1995). This two-step selection was also successfully used in producing transgenic sugarcane, rye, wheat, and *Triticale* plants (Bower and Birch, 1992; Vasil et al., 1992; Castillo et al., 1994; Zimny et al., 1995).

### Polymerase Chain Reaction (PCR)

Recovering resistant embryogenic callus and regenerating transgenic plants on selection medium are not sufficient proof to demonstrate stable integration of transgenes into the plant genome. Molecular analyses are necessary to further confirm stable integration of transgenes in plant genome. In this study, DNA from a number of plantlets (originating from few different resistant embryogenic calli clumps) were obtained and subjected to PCR analyses. DNA from untransformed plants was also isolated and used as negative controls. PCR amplification of an oil palm internal control fragment was carried out prior to the PCR amplification of transformed genes. Based on previous report, the use of a specific pair of primers (POR12 and POR38) will specifically amplify a ∼1.1 Kb size fragment of the oil palm genomic DNA (Nurfahisza et al., 2014). As such all samples used in this study, including the negative controls, were tested for their ability to amplify the 1.1 Kb fragment. Samples failing to amplify were further purified or DNA was re-extracted prior to the amplification of the transgenes.

**FIGURE 4:**
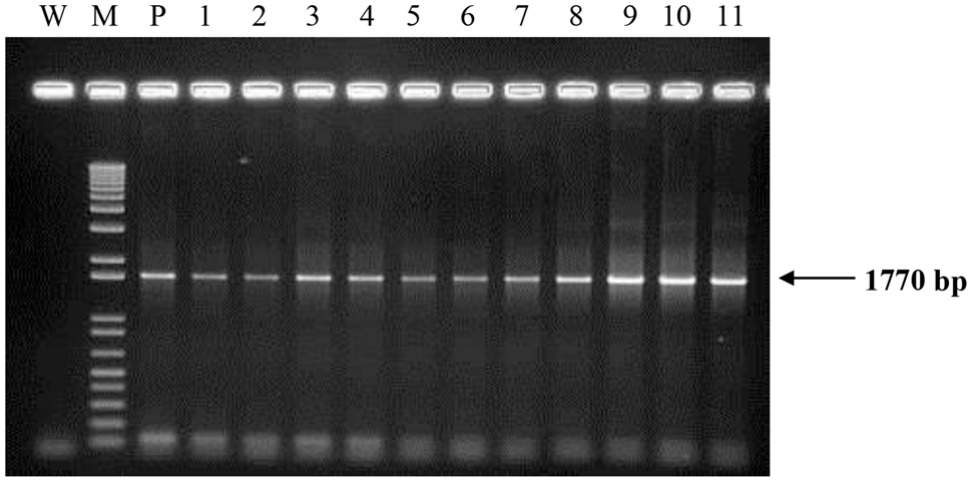
**Polymerase chain reaction (PCR) analysis on DNA from transformed oil palm plantlets using primers for *phaC* gene.** The expected size (1770 bp) is indicated by an arrow. Lane M = 1 kb plus DNA marker; W, water (negative control); P, plasmid control (pME22); 1–11, oil palm samples transformed with *PHB* genes.

All transgenic plantlets regenerated were derived from embryogenic calli which were selected on herbicide Basta after being bombarded with the plasmid carrying *bar* gene. Therefore, amplification of the *bar* gene was used to verify the transformants. Using the *bar* gene primers and a touchdown protocol, a 460 bp amplicon was expected. More than 90% of the transgenic samples tested showed the amplification of the expected size band indicating the presence of the *bar* gene. Samples that were positive for *bar* gene were considered to be putative transgenic and most likely to carry the PHB transgenes. These were therefore later subjected to amplification of the three PHB genes. For these three genes, primers were designed to encompass almost the entire sequence of the genes used for transformation. For the amplification of *bktB* gene a fragment size of 1185 bp was expected, while for the *phaB* and *phaC* genes, fragment sizes 741 bp and 1770 bp, respectively, were expected (**Figure 4**). Most of the tissues tested showed the amplification of the three genes, and for the negative controls no amplification of the genes was observed. A summary of the number of samples analyzed and the results obtained is shown in **Table 1**. Overall, 77 of the *bar* positive samples showed co-integration of the *bar* gene with the other three PHB genes which were not involved in selection. The high frequency of co-integration is not surprising since all genes were linked on the same transforming vector. This high percentage of transgene co-integration is in agreement with the findings reported for soybean and wheat (Christou and Swain, 1990; Vasil et al., 1991). Another observation was that not all the transgenic palms carried the three PHB genes, were some had one or two only, while others appear to be escapes. Similar results were also observed in transgenic rice, alfalfa and switchgrass (Saruul et al., 2002; Endo et al., 2006; Somleva et al., 2008).

**Table 1:** Summary of polymerase chain reaction (PCR) analysis on 56 transgenic plantlets samples transformed with PHB genes.

	**Samples**	**POR^a^**	***Bar*^b^**	***bktB*^c^**	***phaB*^d^**	***phaC*^e^**
pME22	56	56	52	40	49	47
Percentage (%)	100	100	93	77	94	90

*^a^Total number of samples analyzed by PCR and positive for internal control (POR).*

*^b^Total number of samples that was PCR positive for bar gene.*

*^c,d,e^Total number of samples positive by PCR for *bktB*, *phaB*, and *phbC* genes (%). Percentages of samples showing the presence of *bktB, phaB* and *phbC* genes was based on 52 samples that were PCR positive for *bar* gene.*

### Southern Blot Hybridization

**FIGURE 5:**
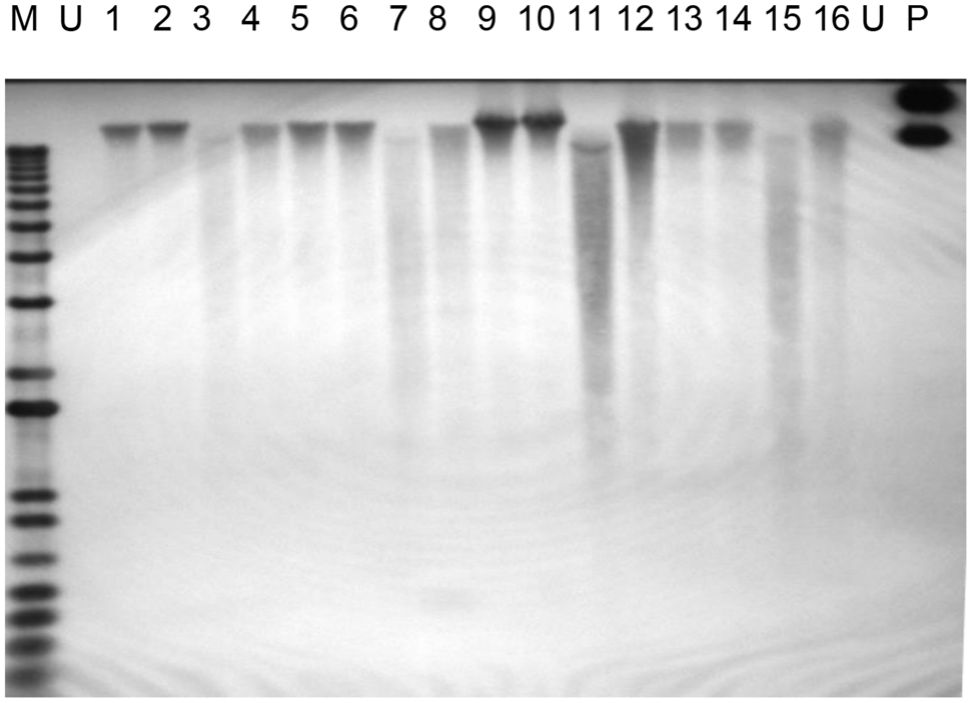
**Southern hybridization on undigested genomic DNA from transformed oil palm using *bar* gene fragment as the probe.** Lane M = 1 kb plus DNA ladder marker; U, untransformed oil palm (negative control); P, plasmid control (positive control pME22), 1–16, oil palm samples transformed with PHB genes.

Polymerase chain reaction analysis of the PHB and selectable marker genes provide initial evidence of the presence of the transgenes in the genome of putative transgenic oil palm. As observed by Potrykus (1990), positive PCR amplifications are not a definitive evidence of stable integration of transgenes into the plant genome, and there are other requirements to confirm the stable integration of a transgene. One of the requirements is Southern blot analysis utilizing high molecular weight genomic DNA. In this study, when restriction digested genomic DNA from transformed oil palm samples was hybridized with the transgenes, very weak signals were obtained (data not shown). This was probably due to non-optimization of hybridization conditions. In cases where undigested DNA samples were hybridized with the *bar* gene (361 bp), hybridization with high molecular genomic DNA was observed (**Figure 5**). Hybridization to the high molecular weight undigested genomic DNA indicates the integration of the transgenes into the oil palm genome. It was also observed that there was no hybridization with undigested total DNA from untransformed sample. In this study a shorter fragment of *bar* gene was used (361 bp) instead of full length (∼600 bp) to avoid some area of the *bar* gene that share some homology with oil palm genome and occasionally resulting in false positive signals. As such, PCR and Southern blot hybridization with undigested DNA have proven the stable integration of the transgenes into the genome of transformed oil palm plantlets.

### Total RNA Isolation and Real-time PCR Analysis

Total RNA from oil palm leaves was successfully isolated based on method by Zeng and Yang (2002) which gave consistent yield of ∼60–120 μg of total RNA from one gram of leaf tissue. The purity of the RNA samples was also good as the A_260_/_280_ ratios were between 1.8 and 2.0. Real-time PCR was carried out to study the expression of *bktB*, *phaB*, and *phaC* genes on 35 putative transgenic samples carrying PHB genes. The RNA from the untransformed plant was used as the calibrator. Removal of DNA contamination from total RNA samples was carried out using the QIAGEN RNase-free DNase set according to the manufacturer’s protocol. RNA integrity is very important in gene expression studies involving real-time PCR. Therefore, only RNA samples with RNA integrity number (RIN) value greater than 5.0 were used to synthesize cDNA. RIN value was determined using the Agilent 2100 Bioanalyzer. The cDNA was later synthesized using the High Capacity cDNA Archive kit (Applied Biosystems). A total of 45 ng of cDNA were used in the RT-PCR analysis. The RQ of the genes was carried out using the comparative ddC_T_ method (Livak and Schmittgen, 2001). Glyceraldehyde-3-phosphate dehydrogenase (*GAPDH*) gene was used as the internal (endogenous) control.

**FIGURE 6:**
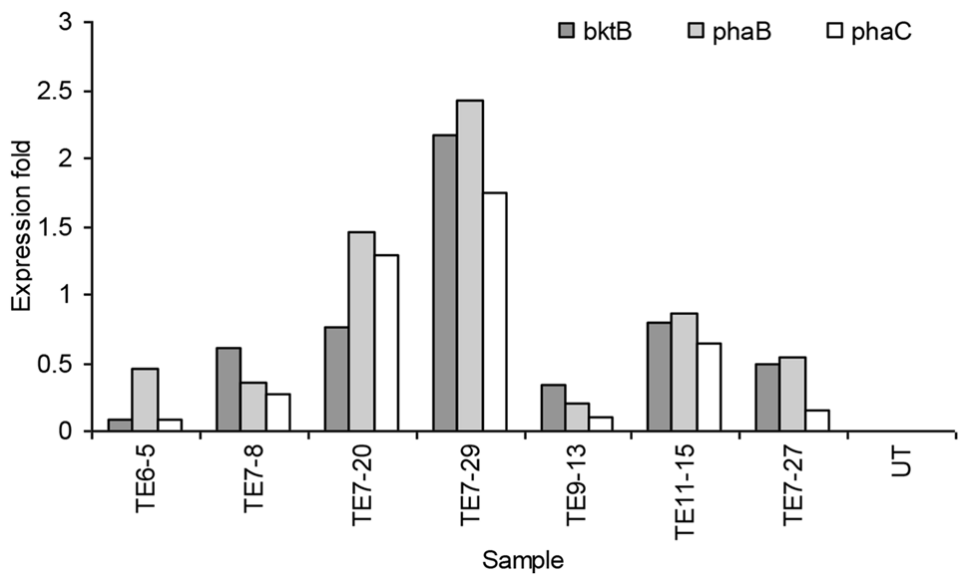
**Expression of *bktB, phaB*, and *phaC* genes in transgenic oil palm determined by real-time PCR analysis.** UT represents the untransformed oil palm (calibrator).

Real-time PCR results showed that not all genes were expressed in the samples. Lower C_T_ (threshold cycle) values reflected more target gene transcripts in the sample and a higher C_T_ indicated less target gene transcripts. Of the 35 transformed plants tested, only seven samples expressed all the three PHB genes. Most of the samples expressed either two or only one of the transgenes. The expression level of all the three PHB genes in the seven samples is shown in **Figure 6**. The highest expression level for all genes was detected in transformant event no TE7-29. The expression was 2.17, 2.42, and 1.75-fold higher than the calibrator for the *bktB*, *phaB*, and *phaC* genes, respectively. Overall, these results indicated that the genes were expressed at low levels in the transgenic plants. It was reported that comparing to GADPH gene as a control, a low expression level of endogenous carotenoid genes was also observed in oil palm (Rasid et al., 2007). The transcript level of carotenoid genes seemed to be lower than the transcript level of the GADPH gene. It was also observed that not all plants that were resistant to Basta also carried and expressed the PHB genes. It is quite common in transformation studies for such phenomenon to occur. It has been reported in tobacco, alfalfa, and rice that not all plants with positive transgene Southern hybridization expressed the PHB genes (Nakashita et al., 2001; Saruul et al., 2002; Endo et al., 2006). This observation could also be due to transgene truncation or silencing as it is a common phenomenon in development of transgenic plants (Meyer and Saedler, 1996).

### Detection of PHB in Transgenic Oil Palm

**FIGURE 7:**
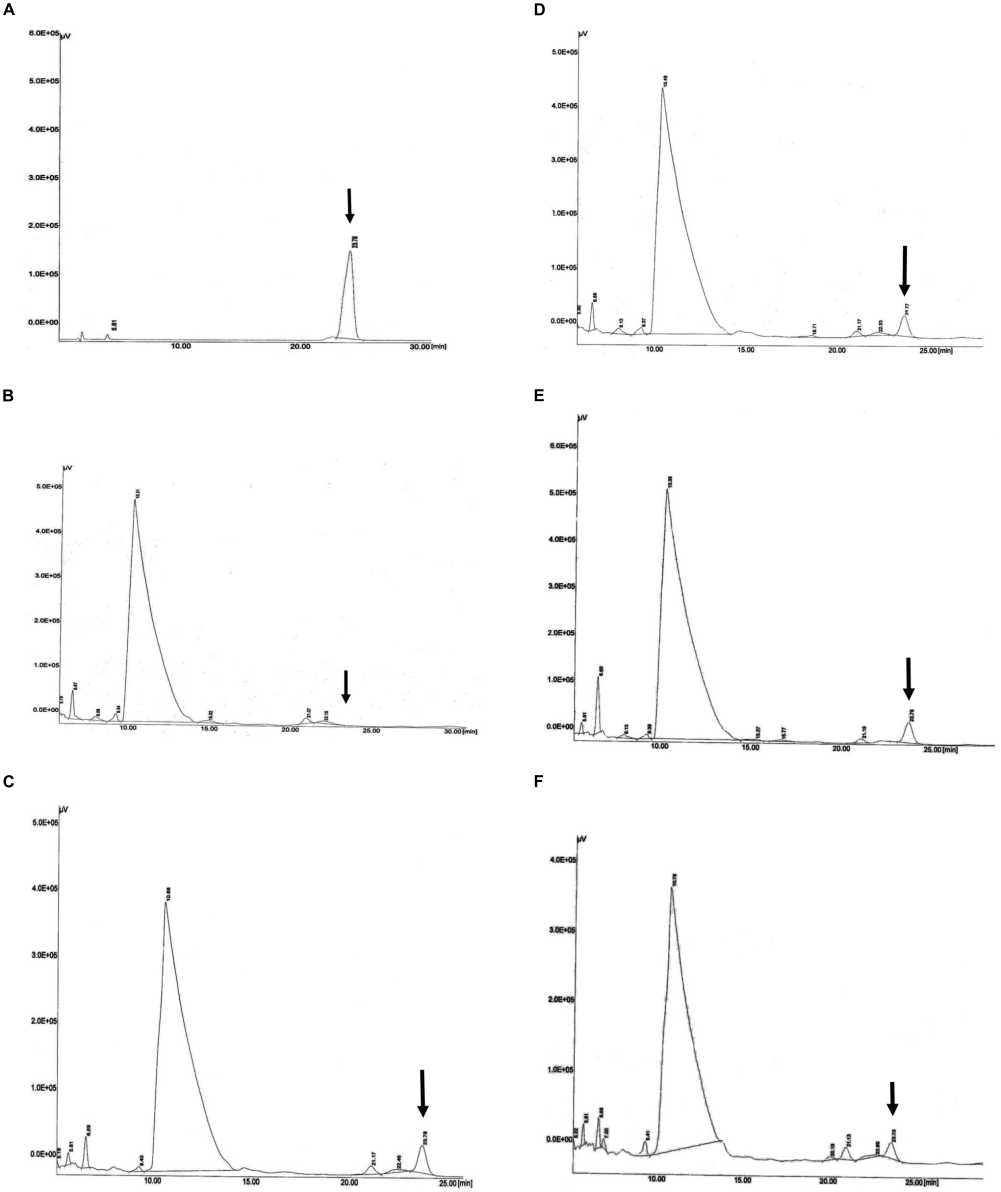
**Detection of PHB in the form of crotonic acid by HPLC. (A)** PHB standard as positive control; **(B)** untransformed oil palm (negative control); **(C–F)** transformants number TE9-4, TE7-28, TE7-29, and TE11-2, respectively, which shows crotonic acid peak at retention time of ∼23.78 min. Arrow indicates crotonic acid retention time.

Detection of PHB in the transformed oil palm was carried out using HPLC. The presence of the PHB in the samples was measured using an acidic methanolysis and hydrolysis method according to Karr et al. (1983). The sulfuric acid used in the methanolysis hydrolyzed the hydroxybutyrate into crotonic acid and water. The crotonic acid was detected as a peak in the HPLC chromatogram. In this experiment, standard (commercial) crotonic acid and acid hydrolyzed commercial PHB standard were used as positive controls. In addition, to confirm the detection of the crotonic acid in oil palm samples, standard crotonic acid, and commercial PHB standard were spiked into untransformed oil palm samples. As expected, a crotonic acid peak was detected in all of the standards (**Figure 7**) and spiked samples. When transformed oil palm samples were subjected to acid hydrolysis and HPLC analysis, PHB was detected in 11 out of 36 samples tested (**Figure 7**). The amount of PHB produced in transgenic oil palm samples were calculated based on the regression equation derived from known crotonic acid and PHB standards used in this study. Based on the known standard concentrations, the concentration of PHB in all samples were calculated. The amount of PHB produced during HPLC analysis was later used to calculate the amount of PHB produced per dwt of oil palm leaves.

**FIGURE 8:**
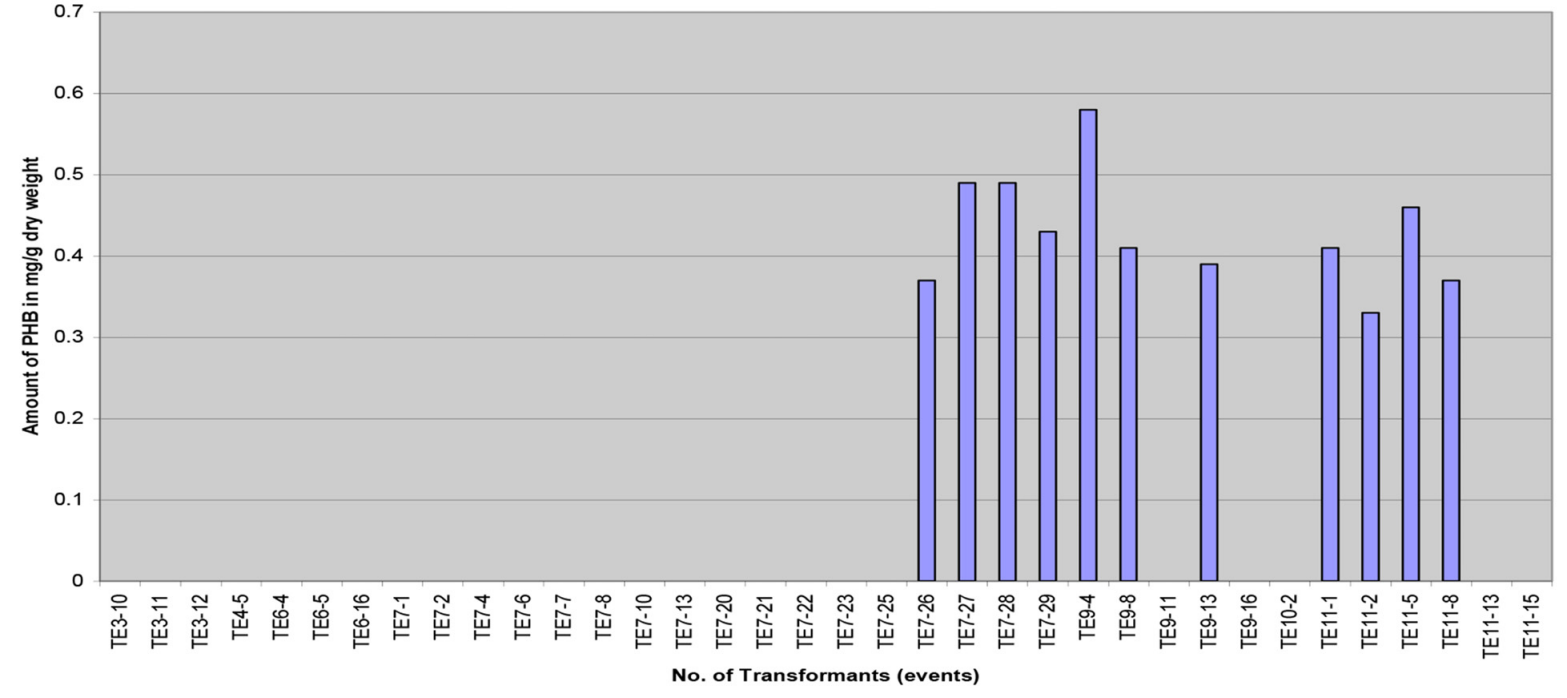
**Polyhydroxybutyrate content in different transformants (transformation events) measured in mg/g of dry weight**.

The amount of PHB obtained was quite low, the content ranging from 0.033 to 0.058% dwt (0.33 to 0.58 mg/g dwt) with an average of 0.043% dwt (**Figure 8**). The determined PHB peak positions of the samples were based on the retention time of crotonic acid, acid hydrolyzed PHB and spiked standards. The results indicate that the amount of PHB obtained was low. It is hoped that when the transgenic palms mature, higher accumulation of PHB would likely be observed as the substrate for PHB, acetyl-CoA, is abundant in the oil palm fruit (mesocarp).

**FIGURE 9:**
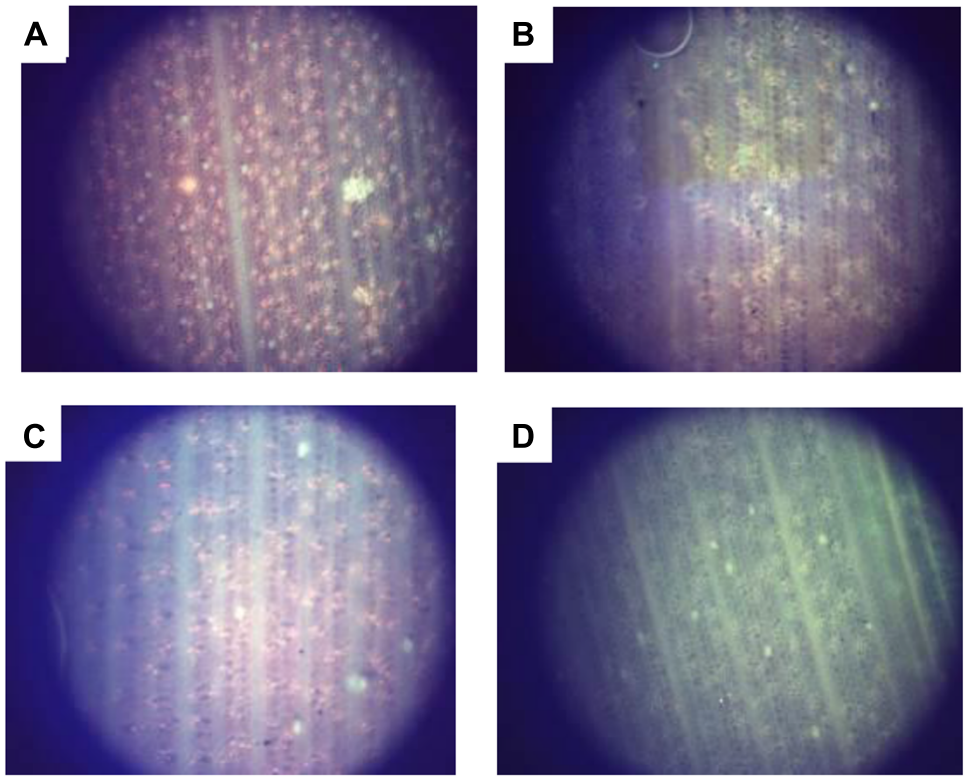
**Accumulation of PHB granules in leaf surface of transformants number TE7-27 **(A)**, TE7-28 **(B)**, and TE7-29 **(C)** stained with Nile blue A.** No PHB granule was observed for untransformed oil palm leaf **(D)**.

To further confirm the PHB content in transformed oil palms, the leaf of TE7-27, TE7-28, and TE7-29 were stained with Nile blue A (**Figure 9**). All leaf samples showed foci of orange fluorescence with different distribution patents. Orange fluorescence was not observed in the leaf of untransformed oil palm stained with Nile blue A indicating that the foci of orange fluorescence observed were due to the presence of PHB granules. Nile blue A staining is the easiest way to visualize PHB granules in cells which were already demonstrated in transgenic plants producing PHB, such as *Arabidopsis* (Nawrath et al., 1994b) and sugarcane (Petrasovits et al., 2007; Purnell et al., 2007). The property of Nile blue A to bind PHB granule resulted in the strong orange fluorescence upon excitation at wavelength 460 nm. Furthermore, cell membranes or other lipid-containing cell components do not absorb Nile blue A to give detectable fluorescence.

Evaluation in the biosafety nursery did not demonstrate any negative effects which could be attributed to the accumulations of PHB. It is postulated that the normal appearance of the transgenic oil palm was due to the low amount of PHB synthesized in oil palm samples tested. It was reported in several plants that a high amount of PHB results in detrimental effect on the growth or morphology of the plants. In flax, it was reported that transgenic plants that produced lower amount of PHB (0.0005–0.0046% FW) demonstrated no negative effects on growth as compared to plant lines producing high PHB content (up to 0.005% FW), which show significant growth reduction and senescence soon after reaching a height of a few centimeters (Wróbel et al., 2004). Similarly in cotton, transgenic plants accumulating a low amount of PHB, from 0.003 to 0.344% dwt fiber and showed normal growth and morphology (John and Keller, 1996). Alfalfa and sugarcane leaves which synthesized PHB up to 0.18 and 1.88% dwt, respectively, were also shown not to have any obvious deleterious effects (Saruul et al., 2002; Petrasovits et al., 2007). Previously, it was shown that PHB can be synthesized up to 1.0% dwt in *Arabidopsis* plastids of presenescing leaves without any negative effects on growth (Nawrath et al., 1994b). However, in a later study, where fully expended leaves were examined, *Arabidopsis* plants that accumulated 0.3% FW of PHB were shown to have a reduction in growth (Bohmert et al., 2000). In addition, all the plants that produced 0.3% FW PHB or higher also showed chlorosis of their leaves. Finally, in the lines that produced the highest amount of PHB, 3.4 and 4.2% FW, the growth was severely stunted and the plants failed to produce any seeds.

There are three possible reasons why the strategy utilized in this study failed to yield high amount of PHB in oil palm leaves. First, all the PHB and selectable marker genes were driven by the same maize ubiquitin promoters. In alfalfa, the presence of four CaMV 35S promoters driving the expression of the PHB and selectable marker genes led to T-DNA rearrangements or gene silencing, which subsequently resulted in low PHB yield (Saruul et al., 2002). Second, the extraction method was probably not very efficient for extracting PHB synthesized in the oil palm leaves. Mittendorf et al. (1998) reported that ∼60–75% of the PHB remained in the *Arabidopsis* plant material after the extraction procedure. It is possible that the PHB detected in the oil palm leaves accounted only for a fraction of PHB polymers that were able to pass through the cell wall matrix; while PHB remaining trapped inside the cells may have escaped HPLC detection. The third reason why the above strategy failed to yield high PHB in the oil palm leaves may be because the PHB was extracted and quantified from the leaves and not from the fruit, where the maximum pool of Acetyl-CoA is present. It was reported that rapeseed contains up to 40% oil per dwt in its seeds. Thus, the seeds should be the most suitable place to produce PHA. This strategy not only avoided deleterious effects on the plant growth but also favored PHB extraction (Liang et al., 2000). Therefore, it is expected that when the transgenic oil palm matures, large amounts of PHB could possibly be produced in its fruits.

## Author Contributions

GKAP conceived and designed research. AK, BB, NHA, MYAM, and OAR conducted experiments. AHT and ZI contributed the research material and advised on some of research activities. GKAP prepared the manuscript. All authors read and approved the manuscript.

## Conflict of Interest Statement

The authors declare that the research was conducted in the absence of any commercial or financial relationships that could be construed as a potential conflict of interest.
